# Application and mechanism of Chinese herb medicine in the treatment of non-alcoholic fatty liver disease

**DOI:** 10.3389/fphar.2024.1499602

**Published:** 2024-11-13

**Authors:** Yuqiao Liu, Yue Fan, Jibin Liu, Xiyang Liu, Xiuyan Li, Jingqing Hu

**Affiliations:** ^1^ College of Basic Medical Sciences, Chengdu University of Traditional Chinese Medicine, Chengdu, China; ^2^ Xin-Huangpu Joint Innovation Institute of Chinese Medicine, Guangzhou, China

**Keywords:** non-alcoholic fatty liver disease (NAFLD), Chinese herb medicine (CHM), insulin resistance, lipid metabolism disorder, mitochondrial dysfunction, oxidative stress, endoplasmic reticulum stress

## Abstract

Non-alcoholic fatty liver disease (NAFLD) is a chronic liver condition closely associated with metabolic syndrome, with its incidence rate continuously rising globally. Recent studies have shown that the development of NAFLD is associated with insulin resistance, lipid metabolism disorder, oxidative stress and endoplasmic reticulum stress. Therapeutic strategies for NAFLD include lifestyle modifications, pharmacological treatments, and emerging biological therapies; however, there is currently no specific drug to treat NAFLD. However Chinese herb medicine (CHM) has shown potential in the treatment of NAFLD due to its unique therapeutic concepts and methods for centuries in China. This review aims to summarize the pathogenesis of NAFLD and some CHMs that have been shown to have therapeutic effects on NAFLD, thus enriching the scientific connotation of TCM theories and facilitating the exploration of TCM in the treatment of NAFLD.

## 1 Introduction

Non-alcoholic fatty liver disease (NAFLD) is a growing public health issue worldwide, encompassing a spectrum of liver conditions ranging from simple fatty liver to nonalcoholic steatohepatitis (NASH), which may progress to cirrhosis and hepatocellular carcinoma (Diehl and Day, 2017). In certain parts of the world, the prevalence of NAFLD is thought to reach up to 26.5% (Younossi et al., 2023). Moreover, NAFLD prevalence in China is 32.5% (Teng et al., 2023). NAFLD is closely associated with components of the metabolic syndrome, such as obesity, type 2 diabetes and hyperlipidemia, and its prevalence is rising in tandem with the increasing prevalence of these metabolic disorders (Younossi et al., 2016).

NAFLD presents itself as a syndrome characterized by the accumulation of fat along with hepatocellular steatosis, ballooning degeneration, lobar inflammation, and predominantly fibrosis (Chalasani et al., 2018). The pathogenesis of NAFLD is complex, involving lipid metabolism disorders (Anwar et al., 2023), insulin resistance (Muzurović et al., 2021), inflammatory responses (Tilg and Moschen, 2010), mitochondrial dysfunction (Mansouri et al., 2018), oxidative stress (Karkucinska-Wieckowska et al., 2022), and endoplasmic reticulum stress (Ajoolabady et al., 2023). Currently, the treatment of NAFLD primarily relies on lifestyle modifications, including dietary adjustments and increased physical activity (El-Agroudy et al., 2019; Golabi et al., 2016). Contemporary medical approaches to treat NAFLD include the improvement of insulin resistance and the use of lipid-lowering drugs, antioxidants, and hepatocyte-protective agents, all promoting liver lipid metabolism and accelerating intrahepatic fat transport (Nassir, 2022). Although some drugs have entered clinical trials, to date, no specific drug has been approved for the treatment of NAFLD (Friedman et al., 2018). Therefore, it is urgent to develop specific drugs for the treatment of NAFLD, which will generate significant social and economic benefits.

Chinese herb medicine (CHM), as an integral part of traditional Chinese medicine (TCM), has unique theories and practical experiences in treating chronic diseases (Shi et al., 2020). The CHM approach to treating NAFLD is usually based on the principle of syndrome differentiation and treatment, aiming to harmonize the body’s yin-yang balance. CHM has been used extensively and safely for millennia to treat liver disorders. In recent years, numerous studies have indicated that certain components of CHM have effects such as modulating insulin resistance (Dai et al., 2022), regulating lipid metabolism (Yang J. M. et al., 2019), anti-inflammation (Lan T. et al., 2021) and antioxidant (Fan et al., 2023), offering new perspectives for the treatment of NAFLD.

With “NAFLD” and “Chinese herb medicine” as key words, we searched CNKI, WanFang, VIP, SinoMed, and PubMed database for relevant literature in the last 10 years. By systematically reviewing relevant literature, this review aims to provide a comprehensive analysis of existing research on the treatment of NAFLD with CHM, explore its mechanisms of action, assess its clinical efficacy, and propose future research directions.

## 2 Multiple hit theory of NAFLD

**FIGURE 1:**
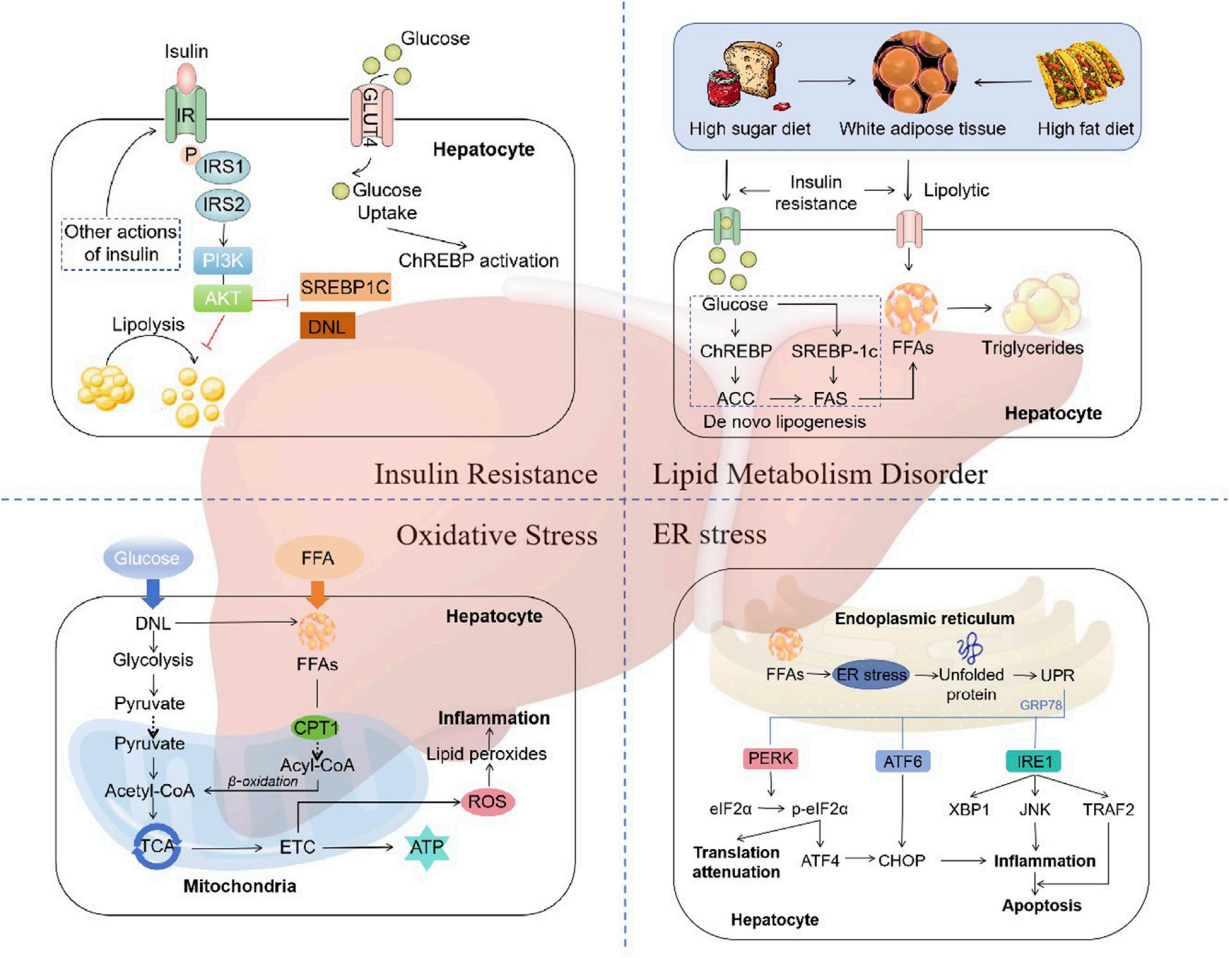
The mechanisms of NAFLD. “Multiple hit” theory includes various factors such as insulin resistance, lipid metabolism disorder, mitochondrial dysfunction, oxidative stress, and endoplasmic reticulum (ER) stress. Abbreviations: IRS, insulin receptor substrate; PI3K, phosphatidylinositol 3-kinase; AKT, protein kinase B; SREBP1C, sterol regulatory element binding protein 1c; DNL, *de novo* lipogenesis; ChREBP, carbohydrate regulatory element-binding protein; ACC, acetyl-CoA carboxylase; FAS, fatty acid synthase; TCA, tricarboxylic acid cycle; ETC, electron transport chain; CPT1, carnitine palmitoyltransferase 1; ROS, reactive oxygen species; UPR, unfolded protein response; PERK, RNA-like ER kinase; eIF2α, phosphorylation of eukaryotic initiation factor-2α; ATF4, activation of transcription factor 4; ATF6, activation of transcription factor 6; CHOP, CCAAT-enhancer-binding protein homologous protein; IRE1, inositol-requiring enzyme 1; XBP1, X-box binding protein-1; JNK, c-Jun N-terminal kinase; TRAF2, TNF receptor-related factor 2.

The traditional “two hit” hypothesis has gradually transitioned to “multiple hit” theory (Fang et al., 2018). In the “two hit” hypothesis, the first hit is represented by insulin resistance associated with obesity, type 2 diabetes, hyperlipidemia, and other conditions, leading to excessive lipid deposition within hepatocytes. The second hit refers to the occurrence of lipid peroxidation and oxidative stress in hepatocytes with excessive lipid deposition, leading to mitochondrial dysfunction, production of inflammatory mediators, and activation of hepatic stellate cells, thereby resulting in NASH and fibrosis (James and Day, 1998). However, as it is inadequate to explain the several molecular and metabolic changes that take place in NAFLD, the “two-hit” hypothesis is now obsolete. “Multiple hit” theory includes various factors such as insulin resistance, lipid metabolism disorder, mitochondrial dysfunction, oxidative stress, and endoplasmic reticulum (ER) stress, etc (Buzzetti et al., 2016) (Figure 1).

### 2.1 Insulin resistance

Insulin resistance (IR) is one of the “multiple hits” predisposing to the development of NAFLD and progression to NASH (Peverill et al., 2014). Dietary factors are crucial for the development of NAFLD. Typical western diet, which has high consumption of fat, has been associated with IR (Fan and Cao, 2013). During the intake of calories, the insulin reduces the production of glucose in the liver by inhibiting glycogenolysis and limiting the postprandial rise in glucose. However, this feedback mechanism is impaired in individuals with IR, resulting in the continued elevation of hepatic glucose production despite the postprandial glucose increases (Fujii et al., 2020). The status of IR leads adipose tissue unresponsive to the antilipolytic effect of insulin, resulting in triglyceride (TG) hydrolysis and the ultimate formation of free fatty acids (FFAs) and glycerol (Schweiger et al., 2006). Increased lipolysis in adipocytes leads to an increase in circulating FFAs, which further exacerbates steatosis and IR in muscle tissue (Zhang et al., 2014).

The molecular mechanism of IR refers to the impairment of the appropriate downstream effects of insulin signaling in target tissues, such as the liver, muscle, and adipose tissue. Insulin exerts its effects on all cells by binding to its specific receptor, thereby initiating a cascade of intracellular signaling (Khan et al., 2019). Upon insulin binding, the insulin receptor phosphorylates itself and several members of the insulin receptor substrate (IRS) family. The typical IRS signalling pathways include those that are dependent on IRS1 or IRS2, which utilise the activities of phosphatidylinositol 3-kinase (PI3K), phosphoinositide-dependent kinase (PDK) and protein kinase B (AKT), as well as the RAS-extracellular-signal regulated kinase (ERK) pathway (Eckstein et al., 2017). Activation of IRS2 has been demonstrated to function as a regulator of sterol regulatory element binding protein 1c (SREBP-1c), thereby influencing *de novo* lipogenesis (DNL) (Schreuder et al., 2008). In states of IR, there is a reduction in the expression of IRS-2, which results in an increase in the expression of SREBP-1c and a corresponding elevation in the rate of DNL (Stefan et al., 2008). Additionally, the β-oxidation of FFAs is suppressed in states of insulin resistance, thereby further promoting the accumulation of hepatic lipids (Postic and Girard, 2008). Thus, disorders of lipid metabolism due to dysregulation of insulin signalling are key factors in the development and progression of NAFLD.

### 2.2 Lipid metabolism disorder

The liver plays a distinctive role in lipid metabolism, acting as a site for lipid uptake, synthesis, oxidation, and distribution of lipids to peripheral tissues (Nguyen et al., 2008). Hepatic fat accumulation results from an imbalance between lipid acquisition and lipid disposal. When energy intake is higher than consumption, excess energy is stored in the form of lipids. In patients with NAFLD, fat accumulates primarily in the form of TG within the liver (Jacome-Sosa and Parks, 2014). The formation of TG is the result of the esterification of glycerol and FFAs. Hepatic steatosis is initiated by an increase in the synthesis of TG in hepatocytes. This synthesis is dependent on the supply of a substrate originating from white adipose tissue (WAT), DNL, and the consumption of a high fat and/or high sugar diet (Donnelly et al., 2005; Heeren and Scheja, 2021; Machado and Cortez-Pinto, 2014).

Dysregulation of DNL is a central feature of liver lipid accumulation in NAFLD patients (Ipsen et al., 2018). The transcriptional regulation of DNL is mainly orchestrated by two key transcription factors: sterol regulatory element-binding protein 1c (SREBP1c) and carbohydrate regulatory element-binding protein (ChREBP) (Eberlé et al., 2004; Sanders and Griffin, 2016). SREBP1c expression is enhanced in NAFLD patients, with higher levels of hepatic triglyceride and upregulating genes coding for acetyl-CoA carboxylase (ACC) and fatty acid synthase (FAS) (Higuchi et al., 2008; Kohjima et al., 2007). In addition, SREBP1c indirectly contributes to the development of hepatic insulin resistance, since enhanced lipogenesis and subsequent accumulation of harmful lipid species, such as diacylglycerides, may interfere with insulin signaling (Kumashiro et al., 2011; Ter Horst et al., 2017). ChREBP is a key transcription factor for enzymes in the fructolysis, glycolysis, gluconeogenesis, and DNL pathways (Iizuka et al., 2004; Iizuka and Horikawa, 2008; Kim et al., 2016). Increased glucose concentration activates ChREBP to regulate the expression of ACC1 and FAS, thereby promoting DNL in hepatocytes (Denechaud et al., 2008; Dentin et al., 2006). Thus, A high fat and/or high sugar diet directly affects the DNL pathways, leading to the accumulation of lipid and the development of NAFLD.

### 2.3 Oxidative stress

A number of factors contribute to the occurrence of “multiple hits”, with oxidative stress being considered the primary cause of liver injury and disease progression in NAFLD (Friedman et al., 2018). An increase in FFAs in the liver, which may result from a number of different causes, can lead to the damage of β-oxidation and mitochondrial dysfunction, resulting in inflammation, which leads to oxidative stress (Chen et al., 2020). Reactive oxygen species (ROS) are important mediators of the inflammatory response (Begriche et al., 2006). ROS, which includes superoxide anion radicals (O2·-) and hydrogen peroxide (H2O2), are continuously produced intracellularly as byproducts of energetic metabolism in different types of liver cells (Masarone et al., 2018). Normal levels of reactive ROS act as signalling molecules that regulate a number of essential cellular processes, including metabolism, survival, immune defence, proliferation and differentiation through the modulation of transcription factors and epigenetic pathways (Forrester et al., 2018). A reduction in ROS generation leads to a decline in redox status and, consequently, impairs the ability of cells to perform physiological redox signaling (Zhang et al., 2019). In the case of oxidative stress, however, excessive ROS induce oxidative modifications to macromolecules, including DNA, lipids, and proteins, leading to the accumulation of damaged macromolecules and subsequent liver injury (Serviddio et al., 2013). In most cells, mitochondria are considered the most quantitatively relevant ROS generators (Mansouri et al., 2018). The liver contains between 500 and 4,000 mitochondria per hepatocyte, which collectively occupy approximately 18% of the cell volume (Degli Esposti et al., 2012). In addition to the mechanisms for the pathophysiology of NAFLD described above, multiple mitochondria-associated factors contribute to the development and progression of NAFLD. Such factors include reduced β-oxidation, impaired, ETC and ATP depletion, over-production of ROS, oxidative stress-mediated cell damage, and ultra-structural mitochondrial changes (Begriche et al., 2006; Begriche et al., 2013; Pessayre et al., 2002). These changes in mitochondrial function and structure exacerbate hepatic lipid accumulation and trigger inflammatory and fibrogenic processes, thereby contributing to the development and progression of NAFLD.

### 2.4 Endoplasmic reticulum stress

The main metabolic pathway affected by endoplasmic reticulum (ER) stress is lipogenesis (Flamment et al., 2010). ER stress is implicated in both the development of hepatic steatosis and the progression of NASH. Disruption of ER homeostasis has been observed in liver and adipose tissue of humans with NAFLD (Gentile et al., 2011; Puri et al., 2008). ER stress represents a protective response that restores protein homeostasis through the activation of the unfolded protein response (UPR) (Li W. et al., 2020). The UPR has been linked to lipid biosynthesis, insulin action, inflammation, and apoptosis (Glimcher and Lee, 2009; Hotamisligil, 2010; Kim et al., 2008).

UPR is mediated by three typical ER-resident stress sensors, protein kinase RNA-like ER kinase (PERK), inositol-requiring enzyme 1 (IRE1), and activating transcription factor 6 (ATF6) (Gong J. et al., 2017). These three proximal UPR sensors all regulate lipid storage in the liver (Donnelly et al., 2005). ER stress has been demonstrated to induce apoptosis through the activation of these three sensor dimers and autophosphorylation (Shao et al., 2022). The IRE1-XBP1 and PERK-peIF2α pathways upregulate the adipogenic gene program (Kaufman, 1999). Conversely, the interaction between ATF6, sterol regulatory element-binding protein 2 (SREBP2) and histone deacetylase 1 (HDAC1) can limit adipogenesis (Zeng et al., 2004). In addition, ATF6 upregulates the expression of X-box binding protein-1 (XBP1), which is one of the main regulators of UPR and interacts with the PI3K insulin signaling pathway, with increased nuclear translocation induced by insulin (Park et al., 2010). XBP1 is a crucial transcription factor that regulates the expression of genes encoding the adaptive UPR. The interaction between PI3K and XBP-1 is subject to modulation by both the cellular response to ER stress and the interaction itself (Winnay et al., 2010). Another consequence of UPR is the activation of SREBP-1c pathways, which results in the maintenance of liver fat accumulation and a further exacerbation of ER stress and UPR (Ferré and Foufelle, 2010). Thus, all three proximal UPR sensors, PERK, IRE1α, and ATF6α, can regulate lipid stores in the liver.

## 3 CHM used in treatment for NAFLD

NAFLD is a complex metabolic disorder that often co-occurs with other metabolic conditions such as dyslipidemia, hypertension, and diabetes mellitus (Zhao et al., 2018). In TCM, while there is no direct equivalent term for NAFLD, the concept of “dampness” and “phlegm” is often used to describe conditions that resemble the symptoms and pathophysiological mechanisms associated with NAFLD. NAFLD can be recognized as hepatic syndromes like distention and fullness, phlegm syndrome like turbidity, hypochondriac pain, lump at the left hypochondrium and damp obstruction disease based on its symptoms and pathogenesis (Zhang and Li, 2017).

In long-term clinical practice, CHM has unique advantages in the treatment of NAFLD. Characterized by a multi-herbal composition and multi-target pharmacological effects, CHM is compatible with the complex pathogenesis of NAFLD. As a result, the majority of NAFLD patients have availed themselves of TCM therapies.

Considering the varying progression of NAFLD, it is imperative to select appropriate CHM for treatment at different stages. Initially, therapeutic interventions for NAFLD focus on soothing the liver, regulating the flow of qi, and bolstering the functions of the spleen and stomach. Xiao Yao San is often used to achieve the effect of soothing the liver and strengthening the spleen in clinical practice. As the condition progresses into the middle and later stages, the primary treatment strategies shift towards strengthening the spleen and nourishing the kidneys, invigorating blood circulation to disperse blood stasis, and additionally incorporating measures to clear heat and resolve dampness. Gexiazhuyu Tang with Erchen Tang are used to activate blood circulation to remove blood stasis, and resolve phlegm to disperse nodules. Sijunzi Tang with Jinguishenqi Pillsis are used to tonify the spleen and kidneys (Zhang and Li, 2017). Meanwhile, the selection of clinical prescriptions should be based on the primary symptom, with consideration given to the other symptoms. It is recommended that a slight variation be made in the selected prescriptions, and that a “differentiation treatment” be employed. Based on clinical experience, the common diagnostic treatment of NAFLD is shown in Table 1.

**TABLE 1:** The common diagnostic treatment of NAFLD.

TCM Syndrome	Main Symptom	Secondary Symptom	Representive Formula	Prescriptions	TCM therapies
Congestion of dampness turbidity	Distension and fullness in the right hypochondriac region	Obesity, general heaviness and fatigue throughout the body, chest and epigastric stuffiness, dizziness, and nausea	Weiling Tang	*Atractylodes lancea (Thunb.) DC [*Asteraceae*; Rhizoma atractylodis], Citrus reticulata Blanco [*Rutaceae*; Tangerine peel], Magnolia obovata Thunb. [*Magnoliaceae*; Magnolia officinalis], Glycyrrhiza glabra L. [*Fabaceae*; Licorice], Alisma plantago-aquatica L. [*Alismataceae*; Rhizoma alismatis], Crotalaria albida B.Heyne ex Roth [*Fabaceae*; Polyporus], Geranium delavayi Franch. [*Geraniaceae*; Red poria], Atractylodes macrocephala Koidz. [*Asteraceae*; Rhizoma atractylodis macrocephalae], Neolitsea cassia (L.) Kosterm. [*Lauraceae*;Cinnamon]*	Dispelling dampness and clarifying turbidity
Stagnation of liver-depression with spleen-deficiency	Distending or migratory pain in the right hypochondriac area, triggered by irritability or anger	Abdominal bloating, loose stools, abdominal pain with an urge to defecate, fatigue, chest distress, frequent sighing	Xiaoyao powder	*Levisticum officinale W.D.J.Koch [*Apiaceae*;Radix angelicae sinensis], Paeonia lactiflora Pall. [*Paeoniaceae*; Radix paeoniae alba], Bupleurum chinense DC. [*Apiaceae*;Radix bupleuri], Smilax glabra Roxb. [*Smilacaceae*; Fuling], Atractylodes macrocephala Koidz. [*Asteraceae*; Rhizoma atractylodis macrocephalae], Glycyrrhiza glabra L. [*Fabaceae*; Prepared liquorice root], Zingiber officinale Roscoe [*Zingiberaceae*; Ginger], Mentha canadensis L. [*Lamiaceae*; Mint]*	Soothing the liver and strengthening the spleen
Accumulation knot of damp and hot	Distending pain in the right hypochondriac region	Nausea, vomiting, jaundice, chest and epigastric fullness, general heaviness, and loss of appetite	Sanren Tang with Yinchen Wuling powder	*Prunus armeniaca L. [*Rosaceae*; Almonds], Talc* [*Mg3(Si4O10) (OH)2*]*, Tetrapanax papyrifer (Hook.) K.Koch [*Araliaceae*; Medulla tetrapanacis], Myristica fragrans Houtt. [*Myristicaceae*; Roud cardamon seed], Zanthoxylum armatum DC. [*Rutaceae*; Bamboo leaves], Magnolia obovata Thunb. [*Magnoliaceae*; Magnolia officinalis], Coix lacryma-jobi L. [*Poaceae*; Coix Seed], Pinellia ternata (Thunb.) Makino [*Araceae*; Rhizoma pinelliae], Artemisia capillaris Thunb. [*Asteraceae*; Herba artemisiae scopariae], Smilax glabra Roxb. [*Smilacaceae*; Fuling], Alisma plantago-aquatica L. [*Alismataceae*; Rhizoma alismatis], Crotalaria albida B.Heyne ex Roth [*Fabaceae*; Polyporus], Neolitsea cassia (L.) Kosterm. [*Lauraceae*; Ramulus cinnamomi], Atractylodes macrocephala Koidz. [*Asteraceae*; Rhizoma atractylodis macrocephalae]*	Clearing heat and transforming dampness
Syndrome of intermin-gled phlegm with blood stasis	Lump or stabbing pain in the right hypochondriac region	Loss of appetite, chest and epigastric oppression, and dull complexion	Gexiazhuyu Tang with Erchen Tang	*Juglans regia L. [*Juglandaceae*; Semen persicae], Paeonia × suffruticosa Andrews [*Paeoniaceae*; Cortex moutan], Paeonia lactiflora Pall. [*Paeoniaceae*; Radix paeoniae rubra], Corydalis turtschaninovii Besser [*Papaveraceae*; Yanhusuo], Glycyrrhiza glabra L.[*Fabaceae*; Prepared liquorice root], Conioselinum anthriscoides ‘Chuanxiong' [*Apiaceae*; Szechuan lovage rhizome], Levisticum officinale W.D.J.Koch [*Apiaceae*;Radix angelicae sinensis], Trogopterori Faeces, Carthamus tinctorius L. [*Asteraceae*; Carthamus tinctorius], Citrus × aurantium L. [*Rutaceae*; Fructus aurantii], Lindera aggregata (Sims) Kosterm. [*Lauraceae*; Radix linderae], Cyperus rotundus L. [*Cyperaceae*; Rhizoma Cyperi], Citrus reticulata Blanco [*Rutaceae*; Tangerine peel], Pinellia ternata (Thunb.) Makino [*Araceae*; Rhizoma pinelliae], Smilax glabra Roxb. [*Smilacaceae*; Fuling], Prunus mume (Siebold) Siebold & Zucc. [*Rosaceae*; Fructus mume], Zingiber officinale Roscoe [*Zingiberaceae*; Ginger]*	Activating blood circulation to remove blood stasis, and resolving phlegm to disperse nodules
Deficiency of spleen and kidney	Right hypochondriac dull pain	Fatigue, sore and weak waist and knees, frequent nocturnal urination, and loose stools	Sijunzi Tang with Jinguishenqi Pills	*Panax ginseng C.A.Mey. [*Araliaceae*; Ginseng], Smilax glabra Roxb.[*Smilacaceae*; Fuling], Atractylodes macrocephala Koidz. [*Asteraceae*; Rhizoma atractylodis macrocephalae], Glycyrrhiza glabra L. [*Fabaceae*; Prepared liquorice root], Rehmannia glutinosa (Gaertn.) DC. [*Orobanchaceae*; Radix Rehmanniae preparata], Cornus officinalis Siebold & Zucc. [*Cornaceae*; Fructus corni], Dioscorea oppositifolia L. [*Dioscoreaceae*; Wild yam], Alisma plantago-aquatica L. [*Alismataceae*; Rhizoma alismatis], Paeonia × suffruticosa Andrews [*Paeoniaceae*; Cortex moutan]*	Tonifying the spleen and kidneys

CHM emphasize on the importance of individualized therapy based on syndrome differentiation, and a variety of CHM formulas are used to alleviate specific symptoms associated with the condition. The selection of formulas is dependent upon the four properties of TCM (cold, hot, warm, cool), the five flavours (sour, bitter, sweet, spicy, salty), and the efficiency. We have assembled a diverse set of CHM formulas that are specifically designed to treat NAFLD and clarified the therapeutic mechanisms by which these CHM formulas ameliorate the disease (Table 2). For example, formulas such as Lingguizhugan decoction, Shenling Baizhu San, Chaihu Shugan powder are commonly used in clinical for the treatment of NAFLD.

**TABLE 2:** CHM prescriptions that are specifically designed to treat NAFLD.

Commercial Chinese polyherbal preparation	Prescriptions	TCM therapies	Indications	Mechanism	Reference
Dang Fei Li Gan Ning Capsules	*Silybum marianum (L.) Gaertn. [*Asteraceae*;Silymarin], Rumex acetosa L. [Polygonacea;Swertia pseudochinensis]*	Clearing damp-heat and benefiting the liver to alleviate jaundice	NAFLD with internal damp-heat retention syndrome	Reducing oxidative stress levels in the liver mediated by nuclear factor E2-related factor 2(Nrf2), promoting liver protein synthesis and bile metabolism, and regulating the expression of transforming growth factor-β1 (TGF-β1) and plasminogen activator inhibitor-1(PAI-1) during disease progression, thereby inhibiting the activation of hepatic stellate cells, collagen proliferation, and promoting the degradation of extracellular matrix, thus preventing liver fibrosis and exerting a hepatoprotective effect	Xiaoling et al. (2022)
Hua Zhi Rou Gan Granules	*Artemisia capillaris Thunb.[*Asteraceae*;Herba artemisiae scopariae], Senna tora (L.) Roxb.[*Fabaceae*;Semen cassiae], Rheum palmatum L. [*Polygonaceae*;Rhubarb], Alisma plantago-aquatica L.[*Alismataceae*;Rhizoma alismatis], Crotalaria albida B.Heyne ex Roth[*Fabaceae*;Polyporus], Crataegus pinnatifida Bunge [*Rosaceae*;Crataegus pinnatifida], Atractylodes lancea (Thunb.) DC[*Asteraceae*;Rhizoma atractylodis], Atractylodes macrocephala Koidz.[*Asteraceae*;Rhizoma atractylodis macrocephalae], Citrus reticulata Blanco[*Rutaceae*;Tangerine peel], Trichosanthes kirilowii Maxim. [*Cucurbitaceae*;Fructus trichosanthis], Ligustrum lucidum W.T.Aiton[*Oleaceae*;Fructus ligustri lucidi], Eclipta prostrata (L.) L.[*Asteraceae*;Herba ecliptae], Lycium barbarum L.[*Solanaceae*;Fructus lycii], Cirsium arvense* var. *arvense [*Asteraceae*;Herba cirsii], Bupleurum chinense DC.[*Apiaceae*;Radix bupleuri], Glycyrrhiza glabra L.[*Fabaceae*;Licorice]*	Clearing heat and resolving dampness, purifying turbidity and detoxifying, and removing blood stasis and softening the liver	NALFD with damp-heat obstructing the middle burner syndrome	Reducing insulin resistance, improving intestinal barrier function, inhibiting endotoxemia, and regulating gut microbiota dysbiosis.Inhibition of macrophage apoptosis and improvement of endoplasmic reticulum stress via the Bcl-2/Bax/Caspase3 signaling pathway	Cao et al. (2024), Liu Y. et al. (2022)
Ke Zhi Capsules	Crustacean shellp [Carapace], Reynoutria multiflora (Thunb.) Moldenke [Polygonaceae; Radix polygoni multiflori], Artemisia capillaris Thunb. [Asteraceae; Herba artemisiae scopariae], Salvia miltiorrhiza Bunge [Lamiaceae; Salvia miltiorrhiza], *Achyranthes aspera* L. [Amaranthaceae; Radix achyranthis bidentatae]	Digesting dampness and turbidity, activating blood circulation and dispersing lumps, and nourishing the liver and kidneys	NAFLD with internal retention of damp turbidity, qi stagnation and blood stasis, or combined with liver and kidney deficiency and depressive heat syndrome	Upregulating the expression of peroxisome proliferator activated receptor γ (PPARγ), insulin receptor mRNA, and protein in liver tissue, and significantly reducing tumor necrosis factor-α (TNF-α), interleukin-6 (IL-6), to exert effects of reducing insulin resistance, regulating fat metabolism, improving liver function, and inhibiting liver inflammation in NAFLD.	Zhao et al. (2014)
Xue Zhi Kang Capsules	*Monascus purpureus Went[*Monascaceae*;Red yeast rice]*	Clarifying turbidity and reducing lipids, activating blood circulation and resolving stasis, strengthening the spleen and aiding digestion	Hyperlipidemia caused by phlegm obstruction and blood stasis	Regulating blood lipids, having anti-inflammatory effect, and exerting protective effect on liver tissue through anti-inflammation mechanism	Zhang Z. et al. (2018)
Xiao Yao Wan (Granules)	*Bupleurum chinense DC.[*Apiaceae*;Radix bupleuri], Paeonia lactiflora Pall.[*Paeoniaceae*;Radix paeoniae alba], Levisticum officinale W.D.J.Koch [*Apiaceae*;Radix angelicae sinensis], Atractylodes macrocephala Koidz.[*Asteraceae*;Rhizoma atractylodis macrocephalae], Smilax glabra Roxb.[*Smilacaceae*;Fuling], Mentha canadensis L.[*Lamiaceae*;Mint], Glycyrrhiza glabra L.[*Fabaceae*;Licorice], Zingiber officinale Roscoe[*Zingiberaceae*;Ginger]*	Soothing the liver and strengthening the spleen, nourishing blood and regulating menstruation	Liver stagnation and spleen deficiency syndrome	Down-regulating PTGS2, up-regulating PPARG, reducing AA content, increasing cAMP, improving insulin resistance, affecting glucose and lipid metabolism, inhibiting oxidative stress and inflammatory response	Ruan et al. (2024)
Hugan Tablets	*Bupleurum chinense DC.[*Apiaceae*;Radix bupleuri], Schisandra chinensis (Turcz.) Baill.[*Schisandraceae*;Schisandra chinensis], Artemisia capillaris Thunb.[*Asteraceae*;Herba artemisiae scopariae], Strobilanthes cusia (Nees) Kuntze [*Acanthaceae*;Radix isatidis], Porcine Gall Bladder Powder, Vigna radiata (L.) R.Wilczek [*Fabaceae*;Green bean]*	Soothing the liver and regulating qi, strengthening the spleen and aiding digestion	Heart and spleen qi deficiency, with phlegm obstruction and blood stasis syndrome	Regulating the levels of MDA, SOD, and GSH-Px in the liver tissue of the model rats, and reversing the metabolic disorders of lipids, sugars, and amino acids	Gong M. et al. (2017)
Gynostemma Pentaphyllum Total Glycosides Tablets	*Gynostemma pentaphyllum (Thunb.) Makino [*Cucurbitaceae*;Gynostemma pentaphyllum]*	Nourishing the heart and strengthening the spleen, tonifying qi and harmonizing blood, expelling phlegm and resolving stasis	Heart and spleen qi deficiency, with phlegm obstruction and blood stasis syndrome	Improving liver function, lipid metabolism, insulin resistance, and levels of inflammatory factors in NAFLD model by regulating LPS/TLR4 signaling pathway	Shen et al. (2020)
Yin Zhi Huang Granules	*Artemisia capillaris Thunb.[*Asteraceae*;Herba artemisiae scopariae], Gardenia jasminoides J.Ellis[*Rubiaceae*;Gardenia], Scutellaria baicalensis Georgi[*Lamiaceae*;Scutellaria baicalensis georgi], Lonicera japonica Thunb.[*Caprifoliaceae*;Lonicera japonica]*	Clearing heat and detoxifying, resolving dampness and reducing jaundice	Damp-heat internal retention syndrome causing elevated ALT levels in both acute and chronic hepatitis	Downregulating protein expression of ACC1 and FASN, and reduce fatty acid absorption by downregulating protein expression of CD36, thereby affecting fatty liver metabolism	Tan et al. (2023)
Silibinin Capsules	*Silybum marianum (L.) Gaertn. [*Asteraceae*;Silymarin]*	Clearing heat and resolving dampness, soothing the liver and promoting gallbladder function	Restoration of liver function abnormalities in patients with acute and chronic hepatitis and fatty liver	Reducing *de novo* lipogenesis and increasing FA oxidation and p-AMPKα expression	Cui et al. (2017)
Fu Fang Yi Gan Ling	*Silybum marianum (L.) Gaertn. [*Asteraceae*;Silymarin], Schisandra chinensis (Turcz.) Baill.[*Schisandraceae*;Schisandra chinensis]*	Nourishing the liver and the kidneys, detoxifying and dispelling dampness	Liver and kidney yin deficiency, with unresolved damp-toxicity syndrome in chronic hepatitis patients with elevated aminotransferase levels	Regulating the immune response and inflammatory reaction in the liver, modulating the liver’s sugar and lipid metabolism, and reducing apoptosis and cell damage in hepatocytes	Guo et al. (2023)

In China, some TCM compounds have been approved as commercial Chinese polyherbal preparation (CCPP), while more TCM compounds are already in clinical trials (Table 3). Such as Dang Fei Li Gan Ning Capsules are primarily indicated for the treatment of patients with non-alcoholic simple fatty liver disease characterized by internal retention of damp-heat (Xiaoling et al., 2022). Hua Zhi Rou Gan Granules are primarily used for the treatment of patients with NAFLD characterized by damp-heat obstructing the middle burner syndrome (Cao et al., 2024; Liu W. et al., 2022). Each type of CCPP is selected based on the specific symptoms and underlying causes identified by a TCM practitioner, ensuring a personalized approach to treatment.

**TABLE 3:** Commonly used commercial Chinese polyherbal preparation (CCPP) for the treatment of NAFLD.

Formula	Prescriptions	Experimental model	Mechanism	References
Lingguizhugan decoction	*Smilax glabra Roxb. [*Smilacaceae*; Fuling], Neolitsea cassia (L.) Kosterm. [*Lauraceae*; Ramulus cinnamomi], Atractylodes macrocephala Koidz. [*Asteraceae*; Rhizoma atractylodis macrocephalae], Glycyrrhiza glabra L.[*Fabaceae*;Licorice]*	C57BL/6J mice with HFD model, Mice bone-marrow-derived macrophages (BMDMs)	Ameliorate HFD-induced hepatic-lipid deposition through inhibiting STING-TBK1-NF-κB pathway in liver macrophages	Cao et al. (2022a)
		Wistar rats with HFD model	Alleviate hepatic steatosis and reduced m6A levels, reduce the m6A methylation levels of suppressor of cytokine signaling 2 (SOCS2), along with the expression of SOCS2 at mRNA and protein levels	Dang et al. (2020)
Zexie–Baizhu Decoction	*Alisma plantago-aquatica L. [*Alismataceae*; Rhizoma alismatis], Atractylodes macrocephala Koidz. [*Asteraceae*; Rhizoma atractylodis macrocephalae]*	C57/BL6 mice with gubra-amylin NASH (GAN) diet-induced NAFLD mouse model	Protect the liver and balance lipid disorders in the NAFLD model via influencing AMPK and Sirt1	Cao et al. (2022b)
Jiangzhi granule	*Gynostemma pentaphyllum (Thunb.) Makino [*Cucurbitaceae*; Gynostemma pentaphyllum], Reynoutria japonica Houtt. [*Polygonaceae*; Rhizoma polygoni cuspidati], Salvia miltiorrhiza Bunge [*Lamiaceae*; Salvia miltiorrhiza], Artemisia capillaris Thunb. [*Asteraceae*; Herba artemisiae scopariae], Glycyrrhiza glabra L. [*Fabaceae*; Licorice]*	C57BL/6J mice with HFD with vitamin D deficiency	Modulate BA profile and activate VDR in HF-VDD-induced NASH mice	Cao et al. (2022c)
Shenling Baizhu San	*Panax ginseng C.A.Mey. [*Araliaceae*; Ginseng], Smilax glabra Roxb. [*Smilacaceae*; Fuling], Atractylodes macrocephala Koidz. [*Asteraceae*; Rhizoma atractylodis macrocephalae], Paeonia lactiflora Pall. [*Paeoniaceae*; Radix paeoniae alba], Lablab purpureus subsp. purpureus [*Fabaceae*; White hyacinth bean], Nelumbo nucifera Gaertn. [*Nelumbonaceae*; Lotus seeds], Coix lacryma-jobi L. [*Poaceae*; Coix seed], Wurfbainia villosa (Lour.) Škorničk. & A.D.Poulsen [*Zingiberaceae*; Fructus amomi], Platycodon grandiflorus (Jacq.) A.DC. [*Campanulaceae*; Platycodon grandiflorus], Glycyrrhiza glabra L. [*Fabaceae*; Prepared liquorice root]*	C57BL/6J mice with western diet + CCl4 injection (WDC)	Ameliorate NAFLD via specific gut microbiota, gut-derived 5-HT, and related metabolites to decrease fat accumulation in the liver and inflammatory responses	Chen et al. (2024)
		Wistar rats with HFD model	Alleviate NAFLD and abnormal lipid metabolism, SIRT1 activation in the liver	Deng et al. (2019)
			Ameliorates NAFLD involves inducing the activation of autophagy, forming a complex regulatory network of key compounds (quercetin, ellagic acid, kaempferol, formononetin, stigmasterol, isorhamnetin and luteolin), key targets (CAT, AKT, eNOS, NQO1, HO-1 and HIF-1α) and related energy metabolites (NADP and succinate), thereby alleviating oxidative stress, ER stress, and mitochondrial dysfunction	Pan et al. (2024)
			Inhibit NLRP3 inflammasome activation and interleukin-1β release by suppressing LPS-induced TLR4 expression in rats with HFD-induced NAFLD	Pan et al. (2021)
Ganshuang granules	*Bupleurum chinense DC. [*Apiaceae*; Radix bupleuri]* *Dioscorea oppositifolia L. [*Dioscoreaceae*; Wild yam], Levisticum officinale W.D.J.Koch [*Apiaceae*; Radix angelicae sinensis], Smilax glabra Roxb. [*Smilacaceae*; Fuling], Atractylodes macrocephala Koidz. [*Asteraceae*; Rhizoma atractylodis macrocephalae]* *Codonopsis pilosula (Franch.) Nannf. [*Campanulaceae*; Codonopsis pilosula], Carapax Trionycis [Turtle shell]* *Taraxacum mongolicum Hand.-Mazz. [*Asteraceae*; Dandelion], Reynoutria japonica Houtt. [*Polygonaceae*; Rhizoma polygoni cuspidati], Prunella vulgaris L. [*Lamiaceae*; Prunella vulgaris], Salvia miltiorrhiza Bunge [*Lamiaceae*; Salvia miltiorrhiza], Juglans regia L. [*Juglandaceae*; Semen persicae]*	C57BL/6J mice with HFD model	Improve liver injury and lipid metabolism disorder by activating the PI3K/AKT signaling pathway, to achieve therapeutic efficacy on NAFLD	Guoguo et al. (2024)
Si Miao Formula	*Atractylodes lancea (Thunb.) DC [*Asteraceae*; Rhizoma atractylodis], Phellodendron amurense Rupr. [*Rutaceae*; Phellodendron amurense], Coix lacryma-jobi L. [*Poaceae*; Coix seed], Achyranthes aspera L. [*Amaranthaceae*; Radix achyranthis bidentatae]*	C57BL/6 mice with high fat/high sucrose (HFHS) diet	Attenuate HFHS diet-induced NAFLD and regulates hepatic lipid metabolism pathways, Modulation of the gut microbiota composition and in particular an increased relative abundance of Akkermansia muciniphila	Han et al. (2021)
Yiqi-Bushen-Tiaozhi Recipe	*Astragalus mongholicus Bunge [*Fabaceae*; Radix astragali], Epimedium brevicornu Maxim. [*Berberidaceae*; Herba epimedii], Smilax glabra Roxb. [*Smilacaceae*; Fuling], Atractylodes macrocephala Koidz. [*Asteraceae*; Rhizoma atractylodis macrocephalae], Reynoutria multiflora (Thunb.) Moldenke [*Polygonaceae*; Radix polygoni multiflori], Crataegus pinnatifida Bunge [*Rosaceae*; Crataegus pinnatifida], Elsholtzia splendens Nakai ex F.Maek. [*Lamiaceae*; Seaweed], Curcuma aromatica Salisb. [*Zingiberaceae*; Turmeric], Juglans regia L. [*Juglandaceae*; Semen persicae]*	C57BL/6J mice with HFD model	Alleviate NASH by regulating the expression of mmu-let-7a-5p, mmu-let-7b-5p, mmu-let-7g-3p and mmu-miR106b-3p miRNAs that potentially modulate inflammation/immunity and oxidative stress	Hong et al. (2020)
Mailuoning Oral Liquid (MLN)	*Lonicera japonica Thunb. [*Caprifoliaceae*; Lonicera japonica], Achyranthes aspera L. [*Amaranthaceae*; Radix achyranthis bidentatae]* *Scrophularia ningpoensis Hemsl. [*Scrophulariaceae*; Radix scrophulariae]* *Dendrobium nobile Lindl. [*Orchidaceae*; Dendrobium]*	C57BL/6 mice with MCD diet model, HepaRG cell	Improved NASH in MCD-fed mice, and the PGC-1α-PPARα signaling pathway was involved in this process	Jia et al. (2022)
Yinchen Linggui Zhugan Decoction	*Artemisia capillaris Thunb. [*Asteraceae*; Herba artemisiae scopariae], Gardenia jasminoides J.Ellis [*Rubiaceae*; Gardenia], Rheum palmatum L. [*Polygonaceae*; Rhubarb], Smilax glabra Roxb. [*Smilacaceae*; Fuling], Neolitsea cassia (L.) Kosterm. [*Lauraceae*; Ramulus cinnamomi], Atractylodes macrocephala Koidz. [*Asteraceae*; Rhizoma atractylodis macrocephalae], Glycyrrhiza glabra L. [*Fabaceae*; Licorice]*	SD rats with HFD model	Reverse the expression levels of TNF-α, IL-6, IL-1β, and NF-κB in liver tissues of NAFLD rats and decrease the expression of inflammatory chemokines CCL2 and CXCL10	Jiang et al. (2022)
Chaihu Shugan powder	*Bupleurum chinense DC. [*Apiaceae*; Radix bupleuri], Citrus reticulata Blanco [*Rutaceae*; Tangerine peel], Conioselinum anthriscoides “Chuanxiong” [*Apiaceae*; Szechuan lovage rhizome], Cyperus rotundus L. [*Cyperaceae*; Rhizoma Cyperi], Citrus × aurantium L. [*Rutaceae*; Fructus aurantii immaturus], Paeonia lactiflora Pall. [*Paeoniaceae*; Radix paeoniae alba], Glycyrrhiza glabra L. [*Fabaceae*; Licorice]*	SD rats with HFD model	Ameliorate NAFLD with IR by decreasing hypertriglyceridemia, hyperglycemia and hyperinsulinemia; up-regulating the mRNA expression of adiponectin and down-regulating the leptin mRNA expression in liver	Jiang et al. (2018)
		C57BL/6L mice with HFD model	Decrease liver inflammation and inhibiting hepatic fatty acid synthesis, inhibiting the TNFα/TNFR1 signaling pathway	Lei et al. (2022)
Qushi Huayu decoction	*Artemisia capillaris Thunb. [*Asteraceae*; Herba artemisiae scopariae], Reynoutria japonica Houtt. [*Polygonaceae*; Rhizoma polygoni cuspidati], Hypericum japonicum Thunb. [*Hypericaceae*; Herba hyperici japonici], Curcuma longa L. [*Zingiberaceae*; Turmeric], Gardenia jasminoides J.Ellis [*Rubiaceae*; Gardenia]*	Wistar rats with HFD model	Improve the structure of the dysfunctional gut microbiota and regulate DG, TG, PA, LPC, LPE and PAF	Ni et al. (2023)
Qushi Huayu granules		C57BL/6 mice with HFD model	Improve hepatic steatosis and corrected the BCAA disorder in NAFLD mice, and the related mechanisms regulated the AMPK/SIRT1/UCP-1 pathway and promoted WAT browning	Zhang et al. (2021)
Qushi Huayu decoction	*Curcuma longa L. [*Zingiberaceae*; Turmeric], Artemisia capillaris Thunb. [*Asteraceae*; Herba artemisiae scopariae], Gardenia jasminoides J.Ellis [*Rubiaceae*; Gardenia], Hypericum japonicum Thunb. [*Hypericaceae*; Herba hyperici japonici], Reynoutria japonica Houtt. [*Polygonaceae*; Rhizoma polygoni cuspidati]*	Wistar rats with MCD diet model	Exerts a hepatoprotective effect against steatosis and fibrosis presumably via depressed MAPK pathways phosphorylation, reinforcement of PPAR-γ and p-p65 translocating into nucleus and enhanced HSCs reprogramming	Lan Q. et al. (2021)
		C57BL/6 mice with HFD model	Inhibit LPS gut-leakage in NASH, which is associated with downregulation of intestinal MAPK pathway	Leng et al. (2020)
			Decreases hepatic DNL by inhibiting XBP1s independent of SREBP1 and ChREBP. Chlorogenic acid, geniposide and polydatin are the potential responsible compounds	Tian et al. (2023)
Huazhi-Rougan formula	*Artemisia capillaris Thunb. [*Asteraceae*; Herba artemisiae scopariae], Senna tora (L.) Roxb. [*Fabaceae*; Semen cassiae], Rheum palmatum L. [*Polygonaceae*; Rhubarb], Alisma plantago-aquatica L. [*Alismataceae*; Rhizoma alismatis], Crotalaria albida B.Heyne ex Roth [*Fabaceae*; Polyporus], Crataegus pinnatifida Bunge [*Rosaceae*; Crataegus pinnatifida], Atractylodes lancea (Thunb.) DC [*Asteraceae*; Rhizoma atractylodis], Atractylodes macrocephala Koidz. [*Asteraceae*; Rhizoma atractylodis macrocephalae], Citrus reticulata Blanco [*Rutaceae*; Tangerine peel], Trichosanthes kirilowii Maxim. [*Cucurbitaceae*; Fructus trichosanthis], Ligustrum lucidum W.T.Aiton [*Oleaceae*; Fructus ligustri lucidi], Eclipta prostrata (L.) L. [*Asteraceae*; Herba ecliptae], Lycium barbarum L. [*Solanaceae*; Fructus lycii], Cirsium arvense* var. *arvense [*Asteraceae*; Herba cirsii], Bupleurum chinense DC. [*Apiaceae*; Radix bupleuri], Glycyrrhiza glabra L. [*Fabaceae*; Licorice]*	C57BL/6J mice with MCD diet model, HepaRG cell	Enhance fecal BA excretion via inhibiting BA transporters, modulates BA profiles, gut dysbiosis as well as the intestinal environment	Li et al. (2022)
Jian Pi Qing Gan Yin decoction	*Atractylodes macrocephala Koidz. [*Asteraceae*;Rhizoma atractylodis macrocephalae], Atractylodes lancea (Thunb.) DC [*Asteraceae*; Rhizoma atractylodis], Artemisia capillaris Thunb. [*Asteraceae*; Herba artemisiae scopariae], Rheum palmatum L. [*Polygonaceae*; Rhubarb], Smilax glabra Roxb. [*Smilacaceae*; Fuling], Sedum sarmentosum Bunge [*Crassulaceae*; Sedum sarmentosum]*	C57BL/6J mice with HFD model	Ameliorate HFD-induced NAFLD in mice by targeting the first and second phases of hepatic steatosis by stimulating the AMPK/PPARα pathway and inhibiting the LXRα/Srebp1/Nf-κb pathway	Liu W. et al. (2022)
Ginseng-plus-Bai-Hu-Tang	*Panax ginseng C.A.Mey. [*Araliaceae*; Ginseng], Plaster of paris[CaSO4·2H2O], Anemarrhena asphodeloides Bunge [*Asparagaceae*; Rhizoma anemarrhenae], Oryza sativa L. [*Poaceae*; non-glutinous rice], Glycyrrhiza glabra L. [*Fabaceae*; Licorice]*	C57BL/6J mice with HFD model	Modulate lipid and carbohydrate metabolism and be able to restore homeostasis	Lu et al. (2020)
Tianhuang formula	*Panax notoginseng (Burkill) F.H.Chen [*Araliaceae*; Panax notoginseng], Coptis chinensis Franch. [*Ranunculaceae*; Rhizoma coptidis]*	C57BL/6J mice with HFD model	Improve NAFLD via the “Lactobacillus-5-MIAA-Nrf2” pathway	Luo et al. (2022)
Shouwu Jiangzhi Granule	*Reynoutria multiflora (Thunb.) Moldenke [*Polygonaceae*; Radix polygoni multiflori], Crataegus pinnatifida Bunge [*Rosaceae*; Crataegus pinnatifida], Curcuma aromatica Salisb. [*Zingiberaceae*; Turmeric], Alisma plantago-aquatica L. [*Alismataceae*; Rhizoma alismatis], Rheum palmatum L. [*Polygonaceae*; Rhubarb], Sinapis alba L. [*Brassicaceae*; White mustard seed], Sedum sarmentosum Bunge [*Crassulaceae*; Sedum sarmentosum]*	C57BL/6J mice with HFD model	Regulated TAGs synthesis to alleviate hepatic lipid accumulation	Qian et al. (2024)
Hedansanqi Tiaozhi Tang	*Panax notoginseng (Burkill) F.H.Chen [*Araliaceae*; Panax notoginseng], Salvia miltiorrhiza Bunge [*Lamiaceae*; Salvia miltiorrhiza], Crataegus pinnatifida Bunge [*Rosaceae*; Crataegus pinnatifida], Nelumbo nucifera Gaertn. [*Nelumbonaceae*; Lotus leaf]*	SD rats with HFD model	Has protective effect against NAFLD *in vitro* and *in vivo* by activating the Nrf2/HO-1 antioxidant pathway	Qiu et al. (2020)
Shenge Formula	*Salvia miltiorrhiza Bunge [*Lamiaceae*; Salvia miltiorrhiza], Pueraria montana* var. *thomsonii (Benth.) M.R.Almeida [*Fabaceae*; Pueraria lobata], Ligustrum lucidum W.T.Aiton [*Oleaceae*; Fructus ligustri lucidi], Atractylodes macrocephala Koidz. [*Asteraceae*; Rhizoma atractylodis macrocephalae], Curcuma aromatica Salisb. [*Zingiberaceae*; Rhizoma wenyujin concisa], Sedum sarmentosum Bunge [*Crassulaceae*; Sedum sarmentosum]*	C57BL/6 mice with HFD model, AML12 hepatocytes	Activated the PPARα signaling by inhibiting ACOX1, which then promoted mitochondrial fatty acid β-oxidation by upregulating CPT1A, therefore inhibiting hepatocyte lipid accumulation and relieving hepatic steatosis	Shang et al. (2024)
San-Huang-Tang	*Rheum palmatum L. [*Polygonaceae*; Rhubarb], Coptis chinensis Franch. [*Ranunculaceae*; Rhizoma coptidis], Astragalus mongholicus Bunge [*Fabaceae*; Radix astragali]*	C57BL/6J mice with HFD model, free fatty acids-induced lipotoxicity in HepG2 cells	Contribute to NAFLD by affecting insulin resistance via activating INSR/IRS1/AKT/FoxO1 pathway	Shi et al. (2022)
Zhenqing recipe	*Ligustrum lucidum W.T.Aiton [*Oleaceae*; Fructus ligustri lucidi], Eclipta prostrata (L.) L. [*Asteraceae*; Herba ecliptae], Dioscorea oppositifolia L. [*Dioscoreaceae*; Wild yam]*	Wistar rats with HFD + STZ model	Ameliorates hepatic gluconeogenesis and lipid storage in diabetic rats induced by HFD and STZ by activating the SIK1/CRTC2 signaling pathway	Song et al. (2020)
Ganjianglingzhu Decoction	*Glycyrrhiza glabra L. [*Fabaceae*; Licorice], Zingiber officinale Roscoe [*Zingiberaceae*; Dried ginger], Smilax glabra Roxb. [*Smilacaceae*; Fuling], Atractylodes macrocephala Koidz. [*Asteraceae*; Rhizoma atractylodis macrocephalae]*	C57BL/6 mice with MCD model	Protect against the development of lean NAFLD by regulating glucose and lipid metabolism, inhibiting the levels of sn-3-O-(geranylgeranyl)glycerol 1-phosphate and lysoPC(P-18:0/0:0) in glycerophospholipid metabolism	Tang et al. (2024)
Danshao Shugan Granule	*Salvia miltiorrhiza Bunge[*Lamiaceae*;Salvia miltiorrhiza], Paeonia lactiflora Pall.[*Paeoniaceae*;Radix paeoniae rubra], Bupleurum chinense DC.[*Apiaceae*;Radix bupleuri], Curcuma aromatica Salisb. [*Zingiberaceae*;Turmeric], Cyperus rotundus L.[*Cyperaceae*;Rhizoma Cyperi], Levisticum officinale W.D.J.Koch [*Apiaceae*;Radix angelicae sinensis], Reynoutria japonica Houtt.[*Polygonaceae*;Rhizoma polygoni cuspidati], Astragalus mongholicus Bunge[*Fabaceae*;Radix astragali], Artemisia capillaris Thunb.[*Asteraceae*;Tarragon], Strobilanthes cusia (Nees) Kuntze [*Acanthaceae*;Radix isatidis], Thlaspi arvense L.[*Brassicaceae*;Herba patriniae], Artemisia argyi H.Lév. & Vaniot[*Asteraceae*;Folium artemisiae argyi], Crataegus pinnatifida Bunge [*Rosaceae*;Crataegus pinnatifida], Raphanus raphanistrum subsp. sativus (L.) Domin[*Brassicaceae*;Radish seed], Reynoutria multiflora (Thunb.) Moldenke[*Polygonaceae*;Radix polygoni multiflori], Schisandra chinensis (Turcz.) Baill.[*Schisandraceae*;Schisandra chinensis]*	260 patients with NAFLD, SD rats with HFD model	Increased activity of superoxide dismutase, a decrease of malondialdehyde as well as reduced NF-κB activity	Wang et al. (2022)
Quzhi Formula	*Reynoutria japonica Houtt.[*Polygonaceae*;Rhizoma polygoni cuspidati], Senna tora (L.) Roxb.[*Fabaceae*;Semen cassiae], Crataegus pinnatifida Bunge [*Rosaceae*;Crataegus pinnatifida]*	C57BL/6 mice feeding a choline-deficient, l-amino acid-defined, high-fat diet	Protected against NASH by inhibiting lipid accumulation, ER stress, and inflammatory responses	Wu et al. (2022)
Diwuyanggan prescription	*Artemisia capillaris Thunb.[*Asteraceae*;Tarragon], Curcuma longa L.[*Zingiberaceae*;Turmeric], Schisandra chinensis (Turcz.) Baill.[*Schisandraceae*;Schisandra chinensis], Rehmannia glutinosa (Gaertn.) DC.[*Orobanchaceae*;Rehmannia glutinosa], Glycyrrhiza glabra L.[*Fabaceae*;Licorice]*	Kunming mice with HFD model	Reduced the HFD-induced disorders of liver function, which were related to steroid hormone biosynthesis, glycerophospholipid metabolism, sphingolipid signaling pathway, fatty acid beta-oxidation, biosynthesis of unsaturated fatty acids, and amino acid metabolism	Xu et al. (2023)
Yinchenhao Tang	*Artemisia capillaris Thunb.[*Asteraceae*;Tarragon], Gardenia jasminoides J.Ellis[*Rubiaceae*;Gardenia], Rheum palmatum L. [*Polygonaceae*;Rhubarb]*	Kunming mice with HFD model	Reduce body weight and improve the lipid metabolism of HFD induced NAFLD. Ameliorate NAFLD by boosting the expression of NR1H4 and APOA1 in both RNA and protein levels	Xu and Cui (2023)
Huangqin decoction	*Astragalus mongholicus Bunge[*Fabaceae*;Radix astragali], Paeonia lactiflora Pall.[*Paeoniaceae*;Radix paeoniae alba], Glycyrrhiza glabra L.[*Fabaceae*;Prepared liquorice root], Ziziphus jujuba Mill.[*Rhamnaceae*;Chinese date]*	SD rats with HFD model	Ameliorate hepatic inflammation in NAFLD rats by blocking the TLR4/NF-κB/NLRP3 pathway, with multi-components and multi-targets action pattern	Yan et al. (2023)
Huangqin-Huanglian Decoction	*Astragalus mongholicus Bunge[*Fabaceae*;Radix astragali], Coptis chinensis Franch.[*Ranunculaceae*;Rhizoma coptidis], Rehmannia glutinosa (Gaertn.) DC.[*Orobanchaceae*;Radix rehmanniae], Gentiana scabra Bunge[*Gentianaceae*;Gentian]*	C57BL/6 mice with HFD model	Alleviate NAFLD in a multi-target way by lowering fatty acids, and decreasing insulin resistance, inflammation, and apoptosis in the liver	Yang et al. (2024)
Hugan Qingzhi medication	*Alisma plantago-aquatica L.[*Alismataceae*;Rhizoma alismatis], Crataegus pinnatifida Bunge [*Rosaceae*;Crataegus pinnatifida], Typha angustifolia L.[*Typhaceae*;Pollen typhae], Nelumbo nucifera Gaertn.[*Nelumbonaceae*;Lotus leaf], Panax notoginseng (Burkill) F.H.Chen[*Araliaceae*;Panax notoginseng]*	Model of hepatic steatosis in the L02 and HepG2 cells	Activate AMPK and PPARα pathways	Yin et al. (2014)
Xiaozhi formula	*Nelumbo nucifera Gaertn.[*Nelumbonaceae*;Lotus leaf], Trichosanthes kirilowii Maxim. [*Cucurbitaceae*;Fructus trichosanthis], Gynostemma pentaphyllum (Thunb.) Makino [*Cucurbitaceae*;Gynostemma pentaphyllum], Benincasa hispida (Thunb.) Cogn.[*Cucurbitaceae*;Chinese waxgourd peel], Salvia miltiorrhiza Bunge[*Lamiaceae*;Salvia miltiorrhiza], Persicaria perfoliata (L.) H.Gross[*Polygonaceae*;Polygonum perfoliatum]*	C57BL/6J mice with HFD model	Attenuate NAFLD by moderating lipid metabolism by activating AMPK and PPAR signaling pathways	You et al. (2024)
Kangtaizhi granule	*Pueraria montana* var. *thomsonii (Benth.) M.R.Almeida[*Fabaceae*;Pueraria lobata], Paeonia lactiflora Pall.[*Paeoniaceae*;Radix paeoniae alba], Hemerocallis citrina Baroni [*Asphodelaceae*;Daylily], Morus alba L.[*Moraceae*;Mulberry leaf], Polygonatum odoratum (Mill.) Druce[*Asparagaceae*;Polygonatum odoratum], Fructus Mori[*Moraceae*;Mulberry], Pseudocydonia sinensis (Dum.Cours.) C.K.Schneid.[*Rosaceae*;Carica papaya]*	SD rats with HFD model, HepG2 cells incubated with 1 mM of FFA	Regulate the AMPK/mTOR signaling pathway	Zhang et al. (2020)
Chai Hu Li Zhong Tang	*Bupleurum chinense DC.[*Apiaceae*;Radix bupleuri], Astragalus mongholicus Bunge[*Fabaceae*;Radix astragali], Pinellia ternata (Thunb.) Makino [*Araceae*;Rhizoma pinelliae], Codonopsis pilosula (Franch.) Nannf. [*Campanulaceae*;Codonopsis pilosula], Atractylodes lancea (Thunb.) DC[*Asteraceae*;Rhizoma atractylodis], Smilax glabra Roxb.[*Smilacaceae*;Fuling], Curcuma longa L.[*Zingiberaceae*;Turmeric], Glycyrrhiza glabra L.[*Fabaceae*;Prepared liquorice root], Zingiber officinale Roscoe[*Zingiberaceae*;Ginger], Ziziphus jujuba Mill.[*Rhamnaceae*;Chinese date]*	SD rats with HFD model, HepG2 cells with 1% long chain fat emulsion	Protects against NAFLD by activating AMPKα, inhibiting ACC activity, down-regulating SREBP2 and HMGR, and up-regulating PPAR-γ	Zhang M. et al. (2018)
Lian-Mei Yin	*Ziziphus jujuba Mill.[*Rhamnaceae*;Chinese date], Coptis chinensis Franch.[*Ranunculaceae*;Rhizoma coptidis], Ophiopogon japonicus (Thunb.) Ker Gawl.[*Asparagaceae*;Radix ophiopogonis], Scrophularia ningpoensis Hemsl. [*Scrophulariaceae*;Radix scrophulariae], Zingiber officinale Roscoe[*Zingiberaceae*;Ginger], Rehmannia glutinosa (Gaertn.) DC.[*Orobanchaceae*;Rehmannia glutinosa], Asini Corii Collap*	C57BL/6 mice with HFD model	Alleviates NAFLD by suppressing Yap1/FOXM1 pathway-mediated lipogenesis, oxidative stress, and inflammation	Zhang et al. (2024)
Chaihu Shugan powder	*Bupleurum chinense DC.[*Apiaceae*;Radix bupleuri], Conioselinum anthriscoides ‘Chuanxiong'[*Apiaceae*;Szechuan lovage rhizome], Citrus × aurantium L.[*Rutaceae*;Fructus aurantii], Citrus reticulata Blanco[*Rutaceae*;Tangerine peel], Paeonia lactiflora Pall.[*Paeoniaceae*;Radix paeoniae alba], Cyperus rotundus L.[*Cyperaceae*;Rhizoma Cyperi], Glycyrrhiza glabra L.[*Fabaceae*;Prepared liquorice root]*	Wistar rats with HFD model	Reduce hepatic lipid accumulation of NAFLD rat model induced by HFD, and its mechanism may be through the action of 15 miRNAs such as miR-34a-5p, miR-146a-5p, miR-20b-5p and miR-142-3p. Reduce the gene and protein expression levels of ACACA, FASN and other fatty acid biosynthesis related enzymes, thus reducing fatty acid biosynthesis	Zheng et al. (2024)

Currently, many clinical workers combined disease differentiation with syndrome differentiation and used proper prescriptions to effectively improve liver function and clinical symptoms in patients with NAFLD, achieving a satisfactory clinical effect.

## 4 Effects and mechanisms of commonly used botanical drugs on NAFLD

In fact, in traditional medicine, botanical drugs play a crucial role in the prevention and mitigation of different human diseases. Due to the complicated metabolites found in botanical drugs, studying the active metabolites became the mainstream of TCM research. According to the literature and clinical experience, the Chinese medicinal herbs commonly used for treatment of NAFLD in clinical practice can be divided into the following types (Table 4). Here, we summarize the pharmacological effects of active metabolites from high-frequency single herbs.

**TABLE 4:** Classification of Chinese medicinal herbs commonly used for NAFLD treatment.

Type	Botanical drugs
Blood-activating and stasis-removing drug	*Salvia miltiorrhiza Bunge[*Lamiaceae*;Salvia miltiorrhiza], Paeonia × suffruticosa Andrews[*Paeoniaceae*;Cortex moutan], Crataegus pinnatifida Bunge [*Rosaceae*;Crataegus pinnatifida], Levisticum officinale W.D.J.Koch [*Apiaceae*;Radix angelicae sinensis], Curcuma aromatica Salisb. [*Zingiberaceae*;Turmeric], Typha angustifolia L.[*Typhaceae*;Pollen typhae], Juglans regia L.[*Juglandaceae*;Semen persicae], Rheum palmatum L. [*Polygonaceae*;Rhubarb], Paeonia lactiflora Pall.[*Paeoniaceae*;Radix paeoniae rubra], Reynoutria japonica Houtt.[*Polygonaceae*;Rhizoma polygoni cuspidati], Rehmannia glutinosa (Gaertn.) DC.[*Orobanchaceae*;Radix Rehmanniae preparata]*
Heat-clearing and dampness-drying drug	*Scutellaria baicalensis Georgi[*Lamiaceae*;Scutellaria baicalensis georgi], Gardenia jasminoides J.Ellis[*Rubiaceae*;Gardenia], Coptis chinensis Franch.[*Ranunculaceae*;Rhizoma coptidis], Artemisia capillaris Thunb.[*Asteraceae*;Herba artemisiae scopariae], Phellodendron amurense Rupr.[*Rutaceae*;Phellodendron amurense], Reynoutria japonica Houtt.[*Polygonaceae*;Rhizoma polygoni cuspidati]*
Spleen-tonifying and qi-supplementing drug	*Atractylodes macrocephala Koidz.[*Asteraceae*;Rhizoma atractylodis macrocephalae], Astragalus mongholicus Bunge[*Fabaceae*;Radix astragali], Citrus reticulata Blanco[*Rutaceae*;Tangerine peel], Codonopsis pilosula (Franch.) Nannf. [*Campanulaceae*;Codonopsis pilosula]*
Aromatic dampness-dispelling drug	*Nelumbo nucifera Gaertn.[*Nelumbonaceae*;Lotus leaf], Atractylodes lancea (Thunb.) DC[*Asteraceae*;Rhizoma atractylodis], Wurfbainia villosa (Lour.) Škorničk. & A.D.Poulsen [*Zingiberaceae*;Fructus amomi], Magnolia obovata Thunb.[*Magnoliaceae*;Magnolia officinalis]*
Spleen-strengthening and dampness-dispelling drug	*Coix lacryma-jobi L. [*Poaceae*;Coix seed], Smilax glabra Roxb.[*Smilacaceae*;Fuling], Atractylodes macrocephala Koidz.[*Asteraceae*;Rhizoma atractylodis macrocephalae]*
Diuresis-inducing drug	*Alisma plantago-aquatica L.[*Alismataceae*;Rhizoma alismatis], Smilax glabra Roxb.[*Smilacaceae*;Fuling]*
Spleen-invigorating for removing food retention drug	*Crataegus pinnatifida Bunge [*Rosaceae*;Crataegus pinnatifida]*
Liver-soothing for qi-regulating drug	*Bupleurum chinense DC.[*Apiaceae*;Radix bupleuri], Curcuma aromatica Salisb. [*Zingiberaceae*;Turmeric], Lycium barbarum L.[*Solanaceae*;Fructus lycii], Mentha canadensis L.[*Lamiaceae*;Mint]*
Liver blood-nourishing drug	Lycium barbarum L. [Solanaceae; Fructus lycii], Levisticum officinale W.D.J.Koch [Apiaceae; Radix angelicae sinensis], Paeonia lactiflora Pall. [Paeoniaceae; Radix paeoniae alba], Reynoutria multiflora (Thunb.) Moldenke [Polygonaceae; Radix polygoni multiflori], Ligustrum lucidum W.T.Aiton [Oleaceae; Fructus ligustri lucidi]
Yin-nourishing and liquid-engendering drug	*Ophiopogon japonicus (Thunb.) Ker Gawl.[*Asparagaceae*;Radix ophiopogonis], Rehmannia glutinosa (Gaertn.) DC.[*Orobanchaceae*;Radix Rehmanniae preparata], Trichosanthes kirilowii Maxim. [*Cucurbitaceae*;Fructus trichosanthis]*
Heat-clearing for liver-calming drug	*Tagetes erecta L. [*Asteraceae*;african marigold], Senna tora (L.) Roxb.[*Fabaceae*;Semen cassiae], Scutellaria baicalensis Georgi[*Lamiaceae*;Scutellaria baicalensis georgi], Morus alba L.[*Moraceae*;Mulberry leaf]*

### 4.1 Shan Zha (*Crataegus pinnatifida* Bunge)

*Crataegus pinnatifida* Bunge and its subspecies *C. pinnatifida* var. *pubescens* Nakai and *C. pinnatifida* var. *Pinnatifida* [syn.: *Crataegus cuneata* Siebold & Zucc.; Rosaceae] yield Shan Zha (crataegi fructus), is recognized for its ability to enhance digestion and alleviate bloating in TCM. Research has shown that *C. pinnatifida* Bunge possesses potent antioxidant and free radical scavenging activities, which can be attributed to its content of various bioactive metabolites, including chlorogenic acid, epicatechin, hyperoside, vitexin, quercetin, rutin, and procyanidins (Barros et al., 2011; Tadić et al., 2008; Zhang et al., 2001). These metabolites are reported to have many pharmacological effects, which are involved in the treatment of various diseases, including hypertension, cardiovascular, anti-oxidative, atherosclerosis, and hyperlipidemiawhich (Bahorun et al., 2003; Kirakosyan et al., 2003; Yoo et al., 2016). Among them, vitexin and quercetin are reported to intervene NAFLD.

The study showed that 5-week vitexin administration (40 mg/kg, i. g.) could obviously reduce hepatic fat deposition, alleviate lipid metabolism, and inhibit liver inflammation in NAFLD mice. In addition, vitexin significantly reduced hepatic macrophage infiltration, obviously downregulated the mRNA and protein expressions of hepatic SREBP-1c, FAS, ACC, and could significantly inhibit the expressions of TLR4/NF-κB signaling in NAFLD mice (Li C. et al., 2020).

Another metabolite, quercetin is also reported to protect the liver. The finding suggested that antiinflammatory responses, antioxidant, and improve ment of lipid metabolism via farnesoid X receptor 1 (FXR1)/Takeda G protein-coupled receptor 5 (TGR5) signaling pathways played key role in the hepatoprotective effects of quercetin (oral gavaged with the quercetin (100 mg/kg) once a day for 8 weeks) in T2DM-induced NAFLD db/db mice (Yang H. et al., 2019). In additional, quercetin revertes the balance of the gut microbiota and counteracted endotoxemia-induced activation of the TLR-4 pathway, subsequently inhibiting the inflammasome response and the activation of the reticulum stress pathway, which resulted in the prevention of deregulation in the expression of lipid metabolism genes (Porras et al., 2017).

In conclusion, *C. pinnatifida* Bunge can exert therapeutic effects on NAFLD through its anti-inflammatory properties, regulation of lipid metabolism, and modulation of the gut microbiota.

### 4.2 Ze Xie (*Alisma plantago-aquatica* L.)

The rhizome of *Alisma plantago-aquatica* L. and its subspecies *A. plantago-aquatica subsp. orientale* (Sam.) Sam. [syn.: *Alisma orientale (Sam.)* Juz.; Alismataceae] yield Ze Xie (alismatis rhizoma), which has been used to treat various ailments, such as dysuria, edema, nephropathy, hyperlipidemia, and diabetes. A wide range of metabolites, mainly triterpenoids, sesquiterpenoids, and diterpenoids, have been isolated from *A. plantago-aquatica* L.; among which the protostane-type triterpenoids, termed alisol A and B have been proved to be effective on NAFLD (Gao et al., 2024; Li and Qu, 2012; Zhang et al., 2017).

Alisol A (100 mg/kg/day, intraperitoneal injection once daily for 4 weeks) effectively attenuated HFD-induced obesity, suppressed hepatic steatosis and improved lipid and glucose metabolism, and improved damaged β-oxidation in DIO mice. The pharmacologic action may be mediated through AMPK/ACC/SREBP-1c pathway activation (Ho et al., 2019). In another study, the MCD-induced mice were simultaneously treated with a daily dose of Alisol A (15, 30, and 60 mg·kg^−1^, ig) for 4 weeks. The results showed that Alisol A can also ameliorate steatohepatitis by inhibiting oxidative stress and stimulating autophagy through the AMPK/mTOR pathway (Wu et al., 2018).

A study indicated that treated with 100 mg/kg Alisol B once daily for 8 weeks showed significant therapeutic effects on DIO + CCl4 and CDA diet-induced murine NASH models. In this study, Alisol B was found to alleviate hepatocyte lipid accumulation and lipotoxicity in NASH mice via regulating RARα-PPARγ-CD36 cascade. Alisol B was also found to relieve cellular ROS level and decrease inflammatory cytokines expression in mouse primary hepatocytes, along with a robust blockade of JNK/NF-κB pathway (Zhao et al., 2022). In another study, NASH was induced in mice fed a MCD diet for 4 weeks. The mice were simultaneously treated with AB23A (15, 30, and 60 mg·kg^−1^·d^−1^, ig) for 4 weeks. The study suggested that Alisol B 23A protects against MCD-induced NASH in mice via activating the FXR signaling pathway, thus decreasing the accumulation of lipids in the liver, hepatic lobular inflammation and pericellular fibrosis (Meng et al., 2017).

In summary, *A. plantago-aquatica* L. may treat NAFLD by regulating pathways related to inflammation and lipid metabolism.

### 4.3 Dan Shen (*Salvia miltiorrhiza* Bunge)

*Salvia miltiorrhiza* Bunge and its subspecies *S. miltiorrhiza* var. *miltiorrhiza* and *S. miltiorrhiza* var. *charbonnelii* (H.Lév.) C.Y.Wu [syn.: *Salvia przewalskii* Maxim.; Lamiaceae] yield Dan Shen (Radix et rhizoma salviae miltiorrhizae), is a traditional and folk medicine in Asian countries, especially in China and Japan (MEIm et al., 2019). *Salvia miltiorrhiza* Bunge is rich in bioactive metabolites such as tanshinones and salvianolic acids, which are believed to exert hepatoprotective, anti-inflammatory, and antioxidant effects (Shi et al., 2019; Wang Y. C. et al., 2015).

It has been demonstrated that tanshinones can modulate multiple targets, including PPARα, CYP1A2, and MMP2, thereby exerting regulatory effects on lipid metabolism, providing antioxidant benefits, and inhibiting fibrogenesis (Hong et al., 2017). Following intraperitoneal injection of tanshinone IIA (10 mg/kg/day) for 2 month, liver steatosis was significantly inhibited in mice on a high-fat diet. This study suggested that tanshinones IIA attenuates oxidative stress by decreasing ROS malondialdehyde (MDA) production and enhancing the activity of total superoxide dismutase (T-SOD) and glutathione peroxidase (GSH-PX), which may contribute to the inhibition of apoptosis and amelioration of liver steatosis (Yang et al., 2017). In addition, HFD-induced rats received 10 mg/kg tanshinone IIA by daily intraperitoneal injection for 3 months, the lipid deposition in the livers of hyperlipidemic rats and modulated the expression of miR-33a and SREBP-2/Pcsk9 signaling pathway proteins was attenuated (Jia et al., 2016).

Administration of Salvianolic acid A (20 and 40 mg/kg BW, respectively) via intraperitoneal injection ameliorates liver steatosis and hepatic damage in high-fat and high-carbohydrate diet-fed mice (Li S. et al., 2020). Additionally, Salvianolic acid A protects the liver from NAFLD caused by a high-fat diet by reducing both hepatic lipid accumulation and inflammation. This anti-inflammatory action may be attributed in part to the modulation of the TXNIP/NLRP3 inflammatory pathways (Ding et al., 2016). Salvianolic acid B is capable of modulating the SIRT1-dependent deacetylation of HMGB1, thereby offering protection against hepatic steatosis and inflammation induced by either a high-fat diet or palmitic acid (Zeng et al., 2015).

Thus, *S. miltiorrhiza* Bunge seemed to play its anti-oxidation role in the treatment of NAFLD.

### 4.4 Chai Hu (*Bupleurum chinense* DC.)

*Bupleurum chinense* DC. [syn.: *Bupleurum scorzonerifolium* Willd. ; Apiaceae] yield Chai Hu (Bupleuri radix), is a herbal medicine for harmonizing and soothing liver qi stagnation (Law et al., 2014). Saikosaponins, especially Saikosaponina and Saikosaponind, as the major bioactive metabolites in *B. chinense* DC., represent anti-inflammatory, anti-oxidant, and hepatoprotective effects to treat NAFLD (Li et al., 2018).

Studies suggest that Saikosaponin a may influence key regulatory pathways involved in lipid metabolism, such as the PI3K/Akt/NF-κB/NLRP3 pathway, which is central to the homeostasis of lipid profiles within the liver (He et al., 2016). Additionally, Saikosaponin d improved lipid homeostasis by coordinately regulating PPARα activation-mediated both inhibition of SREBP1c-dependent FA biosynthesis and induction of FA degradation (Gu et al., 2022). Moreover, Saikosaponin d can induce improvement of fatty liver by decreasing ER-stress-related protein expression (Chang et al., 2021).

Overall, for the effects of *B. chinense* DC. on NAFLD, primary mechanisms are anti-inflammation and oxidative stress.

### 4.5 Jue Ming Zi [*Senna tora* (L.) Roxb.]

*Senna tora* (L.) Roxb. [syn.: *Senna obtusifolia* (L.). H.S.Irwin & Barneby; Fabaceae] yield Jue Ming Zi (Cassiae semen) has long been used for relieving constipation, improving liver function as well as preventing myopia. Anthraquinones, naphthopyranones, naphthalenes, flavones, polysaccharides and other metabolites, have been isolated and identified from Senna tora (L.) Roxb. (Chen et al., 2023).

It was found that *S. tora* (L.) Roxb. ethanol extract effectively inhibited *de novo* lipogenesis and ameliorated hepatic steatosis by promoting AMPK-mediated autophagy (Ding et al., 2023). Another study found that treatment with *S. tora* (L.) Roxb. extract lessened the effects of HFD-induced NAFLD rats, possibly by increasing the activity of antioxidant enzyme, inhibiting the MDA in liver and upregulated the expression of LDL-R to regulate the lipid metabolism process (Meng et al., 2019).

Naphthalenes can reduce lipid accumulation, liver injury and inflammation, gut microbiota disorders, intestinal barrier injury, and metabolic endotoxemia in HFD induced liver injury (Luo et al., 2021).

Oral administration of rhein (150 mg/kg in water) for 40 days significantly increased energy expenditure, reduced body weight, particularly body fat content, improved insulin resistance, and lowered circulating cholesterol levels in DIO mice. Rhein treatment also reduced liver triglyceride levels, reversed hepatic steatosis, and normalized ALT levels in these mice (Sheng et al., 2011). Rhein can reduce the expression of fat mass and obesity-associated protein and simultaneously alleviating oxidative stress and dysregulation in lipid metabolism within HFD rats. This beneficial impact might be linked to the downregulation of key inflammatory markers, such as TLR4, MYD88, and Cyr61 (Lin and Yu, 2021; Qin et al., 2013).

In conclusion,the mechanisms of *S. tora* (L.) Roxb. treating on NAFLD may relate to alleviate oxidative stress, reduce lipid accumulation, anti-inflammation, and regulate gut microbiota disorders.

## 5 Conclusion and perspectives

**FIGURE 2:**
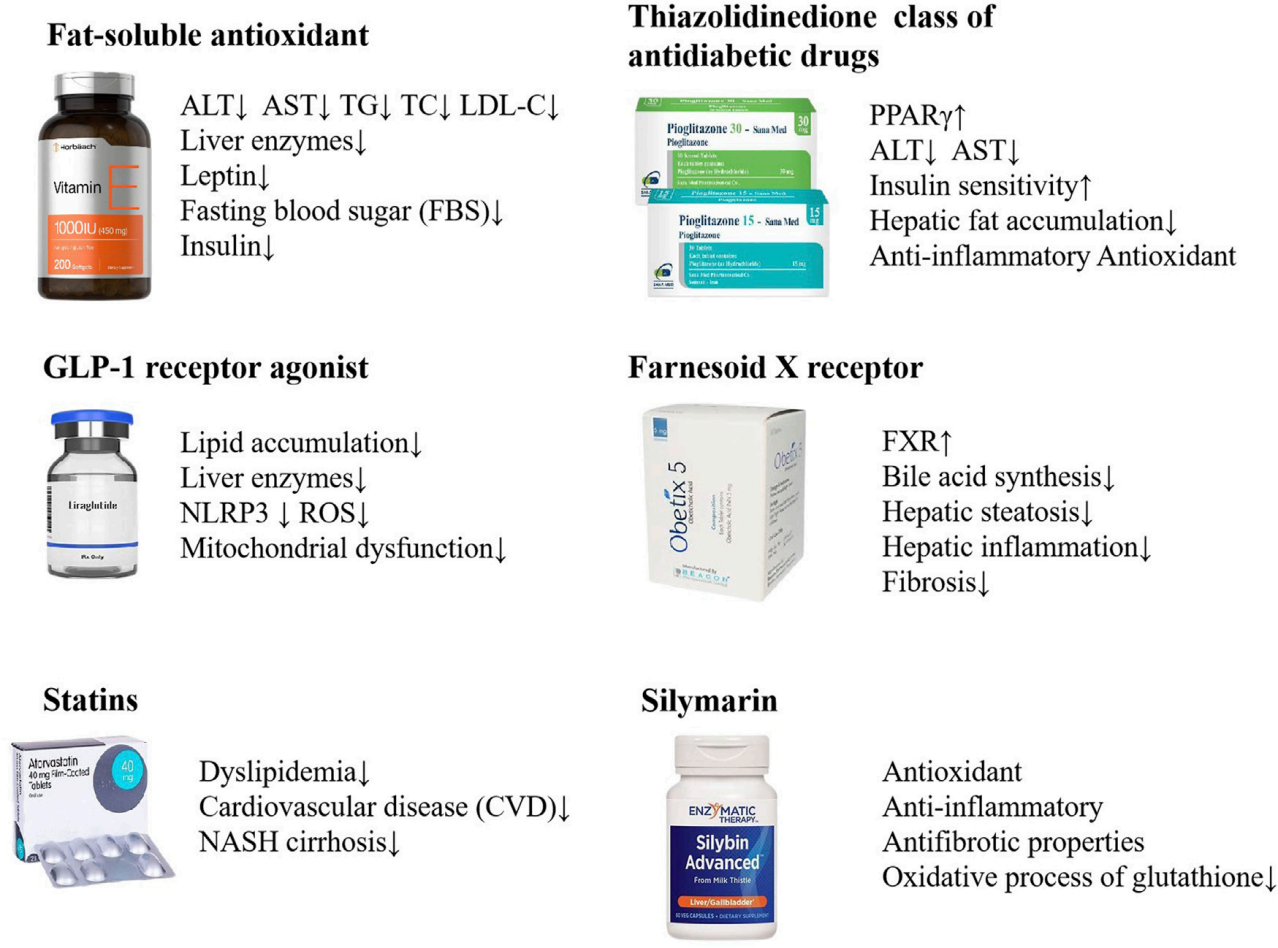
NAFLD treatments with biomedicine. Vitamin E, Liraglutide, Statins, Pioglitazone, Obeticholic acid, Silymarin, etc. are used in the treatment of NAFLD.

The rising global prevalence of NAFLD has become a significant public health issue, necessitating the development of effective and comprehensive treatment strategies. Lifestyle modification with healthy eating and regular exercise represent the primary therapeutic approach for NAFLD (Neuschwander-Tetri, 2017). At present, there are no pharmacological treatments that have been approved by the relevant regulatory authorities for the specific treatment of NAFLD. It is, however, the case that certain pharmaceuticals are efficacious in the treatment of NAFLD, such as insulin sensitizer, lipid-lowering drugs, antioxidants, and weight-loss drugs. These drugs aim to improve metabolic imbalance and liver injury occurred in NAFLD (Dai et al., 2021; van Stee et al., 2018). Vitamin E has been found to correlate with a considerable decrease in liver steatosis, ballooning degeneration of hepatocytes, and pericellular fibrosis (Ekhlasi et al., 2016; Polyzos et al., 2017; Sanyal et al., 2010; Sanyal et al., 2004). The GLP-1 receptor agonist Liraglutide has demonstrated its effectiveness in diminishing the accumulation of fat in the liver and in lowering the levels of liver enzymes among patients diagnosed with nonalcoholic steatohepatitis (NASH) (Newsome et al., 2021; Seino et al., 2010; Yu et al., 2019). Statins can be used in the treatment of dyslipidemia in patients with NAFLD and NASH (Ciardullo and Perseghin, 2021; Pessayre et al., 2002). As a PPARγ agonist, pioglitazone is thought to improve insulin sensitivity in the liver, reduce hepatic fat accumulation, and potentially mitigate liver inflammation and injury through anti-inflammatory and antioxidant actions (Chalasani et al., 2018; Cusi et al., 2016). Obeticholic acid (OCA), a potent ligand for the Farnesoid X receptor (FXR), has been investigated as a therapeutic agent for NASH. Studies have demonstrated that OCA can lead to improvements in the clinical parameters of NASH, including the reduction of fibrosis and the amelioration of liver damage markers. Obeticholic acid (OCA), a potent ligand for the Farnesoid X receptor (FXR), has been investigated as a therapeutic agent for NASH. Studies have demonstrated that OCA can lead to improvements in the clinical parameters of NASH, including the reduction of fibrosis and the amelioration of liver damage markers (Neuschwander-Tetri et al., 2015; Stofan and Guo, 2020). In Europe, Silymarin has been historically uesed as a complementary therapy for the management of hepatic disorders. Silymarin is a key ingredient in the management of liver diseases including chronic hepatitis, cirrhosis, steatosis, alcoholic liver disorders and toxic liver injury, due to its antioxidant, anti-inflammatory and antifibrotic properties (Abenavoli et al., 2010; Schrieber et al., 2011; Wang M et al., 2015) (Figure 2).

CHM, with its principle of “holism” and “individualized treatment,” has demonstrated potential as a multifaceted treatment for the complex pathophysiology of NAFLD. However, CHM has the characteristics of “multicomponents, multitargets, and multipathways,” which also makes it difficult to study. In this review, we summarize the mechanisms of frequently used CCPPs, traditional Chinese formula and single herbs in the treatment of NAFLD. CHM has shown promising therapeutic effects in the treatment of NAFLD by regulating lipid metabolism, reducing inflammation, improving liver function and enhancing antioxidant defenses, with fewer adverse reactions. Thus, the use of CHM in the prevention and treatment of NAFLD has a broad development prospect.

Despite the promising therapeutic effects, there are inherent challenges in CHM treatment for NAFLD. Firstly, most patients with NAFLD do not have obvious symptoms and signs, making the diagnosis of the condition based only on the four diagnostic methods of TCM without objective evidence. Secondly, the CHM in registered clinical trials are limited, because long term effects of CHM are difficult to evaluate. In contrast to numerous synthetic pharmaceuticals, the gradual and nuanced actions of CHM may not manifest immediate outcomes. Consequently, it is essential to conduct comprehensive and prolonged observation periods to fully assess the efficacy and safety profiles. This presents a significant challenge in the design of clinical trials, as they must be sufficiently long-term to accurately assess the true benefits and potential adverse effects of CHM, while also being feasible in terms of resources and patient compliance. Lastly, a comprehensive understanding of the mechanism of CHM on NAFLD are not fully elucidated. The complex interplay of multiple bioactive compounds in CHM presents a great challenge in accurately discerning its precise mode of action. While this complexity is a defining feature of CHM’s holistic approach to treatment, it also necessitates advanced research methodologies to elucidate the complex interactions between CHM components and the biological systems with which they interact. Thus, the application of advanced scientific techniques, including metabolomics, transcriptomics, network pharmacology and microbiome analyses, is essential tol to fully elucidate the therapeutic activities of CHM and to substantiate its mechanisms of action in the context of NAFLD treatment. These approaches can facilitate a more systematic understanding of the underlying mechanisms of TCM, paving the way for future investigations.
